# Comparison of direct laryngoscope and McGrath videolaryngoscope in terms of glottic view and hemodynamics in bariatric surgery

**DOI:** 10.3906/sag-1905-77

**Published:** 2020-02-13

**Authors:** Mehmet ÇAKIR, Erhan ÖZYURT

**Affiliations:** 1 Department of Anaesthesiology and Reanimation, Uzunköprü State Hospital, Edirne Turkey; 2 Department of Anaesthesiology and Reanimation, University of Health Sciences, Antalya Training and Research Hospital, Antalya Turkey

**Keywords:** McGrath videolaryngoscope, bariatric surgery, airway management, glottic view, hemodynamics

## Abstract

**Background/aim:**

In the recent years, videolaryngoscopes (VL) have emerged as alternative devices to direct laryngoscopes (DL) in difficult intubation situations. Therefore, we aimed to compare the Macintosh DL and McGrath VL in terms of the glottic image quality, intubation success, intubation time, hemodynamic response after intubation, and complications in bariatric surgery patients.

**Material and methods:**

After obtaining approval by the ethics committee and receiving informed consent, we recorded the demographic and physical data of patients undergoing bariatric surgery. Patients were divided into 2 groups: Group M was intubated with the Macintosh DL, and Group V was intubated with the McGrath VL. After intubation, we noted the Cormack–Lehane score, the duration of intubation, the number of intubation interventions, and the hemodynamic data of patients.

**Results:**

A total of 62 patients (ASA II, body mass index of >35 kg/m2) were included in the study. All patients except 1 patient were intubated on the first attempt. Although there was a decrease in heart rate and blood pressure with induction, similar hemodynamic data were obtained between groups during the operation. In group V, we obtained a better glottic image (P = 0.011), but intubation success was similar between the study groups. We also measured the intubation time in group M as 45.9 ± 19.1 s and group V as 57.1 ± 15.8 s (P = 0.015).

**Discussion:**

Although we measured longer intubation times with the McGrath VL compared with the Macintosh DL, we obtained a better glottic image without causing hemodynamic changes. However, these findings did not make any difference in terms of intubation success.

## 1. Introduction

Obesity has become an epidemic disease since the 1980s. For patients who cannot lose weight with conservative treatments, such as diet and exercise, surgical options have become prominent in recent years [1]. Bariatric surgery has been performed in an increasing number of individuals since 1991 when it was first announced. Laryngoscopy and intubation may be difficult because of the increase in fat storage in the neck region, changes in the oropharyngeal anatomy, large tongue, and comorbidities of patients undergoing bariatric surgery [2].

Difficult or unsuccessful intubation is one of the major causes of mortality and morbidity in anaesthesia practice [3,4]. Although it is reported that the frequency of difficult intubation in obese patients is high, this issue remains controversial [u28f4]. There is a need for improvements to facilitate intubation and shorten the laryngoscopy time in this patient group [6].

Videolaryngoscope (VL) devices provide a wider glottic image and have emerged in recent years as an alternative to direct laryngoscopy in difficult airway management [7]. The McGrath VL (Aircraft Medical Ltd., Edinburgh, UK) has a handle and a blade similar to the Macintosh direct laryngoscope (DL). There is also a small camera and light source at the end of the laryngoscope blade. The image obtained is transferred to a small screen at the end of the laryngoscope handle. VLs have come to the forefront regarding their ability to visualize anatomical structures and can be effective teaching tools. In addition, laryngoscopy and endotracheal intubation can be performed without any need to keep the oral cavity, pharynx, and larynx aligned axially in cervical spine anomalies and difficult airway conditions. Because of these advantages of VLs, anaesthesiologists argue that it is time to move away from the use of the Macintosh DL, and replace it with more effective devices [8].

The hypothesis of this study was to use VLs as the first choice for endotracheal intubation in patients with the possibility of difficult intubation and determine if it provides an advantage. To test this hypothesis, we aimed to compare the Macintosh DL and McGrath VL regarding the glottic image quality, intubation success, intubation time, hemodynamic response after intubation, and complications in bariatric surgery patients.

## 2. Materials and methods

After the approval by the local ethics committee (Protocol no: 2017-005), the patients who underwent elective bariatric surgery in the ASA II-III classification between the ages of 18 and 65 were included in the study. We did not include patients with a history of a difficult airway, known airway pathology, or who underwent cervical spinal cord surgery in the study. We performed the preoperative evaluation in the anaesthesia clinic and obtained informed consent forms. We recorded age, sex, height, weight, dental condition, thyromental distance, body mass index, and Mallampati scores in the preoperative examination of the patients. Patients were divided into 2 groups as Group M: intubated with Macintosh DL, and Group V: intubated with McGrath VL. A computer-based random number list was used to determine groups. 

In the operating room, standard monitoring including an electrocardiogram, noninvasive blood pressure, pulse oximeter, and capnometer were performed on all patients after they were premedicated with midazolam 1 mg IV. For both groups, anaesthesia induction was provided with propofol at 2–3 mg/kg IV and fentanyl at 1 µcg/kg IV. We used rocuronium at 0.6 mg/kg IV for muscle relaxation. Drugs were administered according to predicted body weight. We ventilated patients with 100% oxygen by a mask in a ramped position and waited for 2 min for adequate muscle relaxation. We intubated patients using the Macintosh DL or McGrath VL at the end of 2 min. We performed the endotracheal intubation in Group V by seeing it from the screen, and in Group M by seeing it directly. We evaluated the laryngoscopic vision by the Cormack–Lehane (C–L) score. All patients were intubated by an anaesthesiologist who was experienced in both devices. For intubation, we used an endotracheal tube with a 7 mm internal diameter for women and 8 mm internal diameter for men. In addition, in Group V, we placed a stylet in all endotracheal tubes. We considered any intubation attempt that lasted more than 3 times or longer than 120 s as a failure. When we encountered such a situation, we planned to withdraw the patient from the study, and continued anaesthesia management based on the ASA difficult airway algorithm. The duration of the intubation period was considered and started from the laryngoscope blade being sent into the mouth until the end-tidal CO2 level was seen. After intubation, we noted the C–L score, the duration of intubation, the number of intubation interventions, and the complications (bleeding, laceration, tooth damage, and other issues). We intubated patients in the supine position and then placed them in the French position. Laparoscopic sleeve gastrectomy operations were performed by the same surgical team under 15 mmHg intraabdominal pressure. We recorded the heart rate, systolic blood pressure, diastolic blood pressure, mean blood pressure, peripheral oxygen saturation of patients before induction (T0), 5 (T1), and 15 (T2) min after intubation, and at the end of surgery (T3). We also recorded the end-tidal carbon dioxide at T1, T2, and T3 times.

### 2.1. Statistical Analysis

The sample size of this study was calculated to detect a difference of 10.1 s in the mean intubation time with a standard deviation of 13.6 s [9,10]. The analysis indicated that 30 patients would be required per group to achieve a power of 80% and an alpha error of 0.05. Considering the possible dropouts, we decided to include 31 patients in each group. For the statistical analysis of our study, SPSS for Windows 16.0.1 (SPSS Inc., Chicago, IL, USA) package program was used. The continuous data are presented as mean ± SD, and categorical data are shown as the number of cases and percentages. The categorical variables were compared using the chi-square test. Continuous variables were compared with the student’s t-test or Mann–Whitney U test according to the distribution of data. A P-value of <0.05 was considered statistically significant.

## 3. Results

A total of 62 patients (31 patients in each study group) were included in the study. All patients were intubated successfully, and no patient was excluded from the study. No laparotomy was performed in any patient. Operations were completed without any complications. 

The demographic and operational data of patients were similar between the groups (Table 1). Peripheral oxygen saturation of all patients was over 97% in laryngoscopy and intubation. Although there was a decrease in the heart rate and blood pressure with induction, we obtained similar hemodynamic data between the groups during the operation (Table 2). 

**Table 1:** Patients characteristics and operation data of study groups.

	Group Mn = 31	Group Vn = 31	P-value
Age (years) (mean ± SD)	39.0 ± 9.8	42.0 ± 10.5	0.246
Sex (M/F) n (%)	3/28 (10/90)	7/24 (23/77)	0.167
Body mass index (kg/m2) (Mean ± SD)	46.5 ± 4.2	46.1 ± 6.6	0.764
Comorbidity n (%)	11 (35.5)	11 (35.5)	-
Thyromental distance (cm) (Mean ± SD)	6.3 ± 0.5	6.1 ± 0.7	0.219
Mallampati (I/II/III/IV)	1/12/16/2	2/11/17/1	0.621
Intubation time (s) (Mean ± SD)	45.9 ± 19.1	57.1 ± 15.8	0.015
Surgery time (min) (Mean ± SD)	79.0 ± 17.9	77.7 ± 21.1	0.796
Anaesthesia time (min) (Mean ± SD)	105.2 ± 17.4	105.3 ± 22.5	0.985

**Table 2:** Hemodynamic parameters of study groups.

	Group M(Mean ± SD)	Group V(Mean ± SD)	P value
Heart rate (beats/min)			
T0	88.1 ± 12.1	86.9 ± 12.6	0.698
T1	82.3 ± 12.1	83.3 ± 12.0	0.745
T2	81.2 ± 11.5	80.9 ± 12.9	0.926
T3	79.5 ± 13.3	83.9 ± 11.8	0.176
Systolic Blood Pressure (mmHg)			
T0	141.9 ± 18.5	140.2 ± 13.5	0.686
T1	124.6 ± 14.3	117.9 ± 15.9	0.088
T2	121.9 ± 16.3	113.5 ± 18.9	0.065
T3	128.5 ± 15.8	128.8 ± 16.3	0.944
Diastolic blood pressure (mmHg)			
T0	80.3 ± 13.3	78.2 ± 13.1	0.549
T1	67.9 ± 15.3	66.9 ± 10.6	0.767
T2	69.8 ± 18.7	66.5 ± 11.5	0.418
T3	69.7 ± 10.1	74.2 ± 10.9	0.099
Mean blood pressure (mmHg)			
T0	105.9 ± 12.1	103.2 ± 11.5	0.372
T1	90.3 ± 13.4	87.7 ± 10.8	0.403
T2	89.6 ± 13.2	85.8 ± 13.0	0.251
T3	92.2 ± 10.5	95.4 ± 11.3	0.264
Oxygen saturation (%)			
T0	98.1 ± 1.4	98.4 ± 1.7	0.303
T1	98.0 ± 1.5	98.0 ± 1.7	0.939
T2	97.8 ± 1.4	97.7 ± 1.8	0.939
T3	98.1 ± 1.6	97.9 ± 1.4	0.568
End-tidal CO2 (mmHg)			
T1	39.0 ± 11.0	35.5 ± 2.1	0.096
T2	38.1 ± 1.9	37.3 ± 2.0	0.175
T3	37.5 ± 2.1	37.1 ± 1.8	0.413

T0: Basal measurement, T1: 5 min after intubation, T2: 15 min after intubation, T3: End of surgery

When we evaluated the patients according to the C–L score, we obtained a better glottic image in Group V (Table 3). In Group M, we achieved intubation on the first attempt in 30 cases, and the second trial in 1 case. We achieved intubation in all 31 cases in Group V on the first attempt (P > 0.05). We measured a longer intubation time in group V compared with group M (group V = 57.1 ± 15.8 s, group M = 45.9 ± 19.1 s, P = 0.015). We did not observe any complications in both groups. 

**Table 3:** Cormack–Lehane scores of study groups.

	Group M n (%)	Group V n (%)	P-value
I- II	9 (29)	19 (61.3)	0.011
III- IV	22 (71)	12 (38.7)	

## 4. Discussion

Bariatric surgery, which is among the treatment options for morbid obesity, is performed more and more every day. In our study of these cases, which are the usual suspects for difficult intubation, we measured longer intubation times with the McGrath VL, but we obtained a better glottic image with the Macintosh DL.

Anatomical and physiological changes that occur in obese patients lead to oxygenation and airway disorders. Adipose tissue increases in pharyngeal structures, narrowing the airway lumen due to the large tongue and mucosal oedema, thus making laryngoscopy and intubation difficult [u28f6]. Here, the C–L classification is a good predictor of the success of laryngoscopy [u28f7,u28f8,u28f9]. However, providing a good glottic image on the VL screen does not necessarily mean that the endotracheal tube will pass through the larynx easily. With the McGrath VL, the image is reflected on the external display via the camera at the tip of the blade. For anaesthesiologists with limited experience, this indirect view may cause a deterioration in hand-eye coordination [16]. Maassen et al. [17] in their study of 150 morbidly obese patients, compared DL with 3 different VLs (GlideScope, V-MAC, and McGrath) and reported that VLs could provide better glottic images and successful intubation without the need for a stylet. Although we obtained a better glottic image in the VL group, we could not detect a difference in the success of intubation.

The decrement of functional residual capacity and lung compliance that occurs in obese patients reduce their tolerance to apnoea periods [u2949]. This situation creates a time pressure on the person who is performing the intubation. Therefore, airway safety in obese patients should be provided as soon as possible. Although it is claimed that the time of intubation with VL is shortened [u294a]. First, there is a difference between the studies concerning the definition of intubation time. In addition, the patient groups in the studies are heterogeneous [u294b]. In a study by Yumul et al. [14], they compared 3 types of VL and DL in obese patients. They found no difference regarding intubation times between the McGrath VL and the Macintosh DL. Arıcı et al. [11] also found an increment in intubation times with the McGrath VL in their study with obese pregnant women. In this study, although the glottic image was better and the anaesthesiologist had experience with the McGrath VL, we found a longer duration of the intubation period in Group V. Another aspect, when compared with the Macintosh, was that a gum elastic bougie is needed more frequently for intubation with the McGrath VL [18]. In our study, we intubated all patients in group V using a stylet. Because of the difficulty encountered in directing the intubation tube, and the time required for the removal of the stylet after intubation, we may have obtained longer intubation times in group V. Nevertheless, we determined that the prolonged intubation time did not cause any negative condition in terms of oxygenation and hemodynamic parameters. This may be because all patients were intubated on the first attempt, except 1 patient, and we performed preoxygenation. 

Contradictory results are obtained in studies comparing the hemodynamic effects of VLs and DLs [19–21]. The underlying cause may be the use of a wide variety of VLs and the different patient populations in the studies. In our study, when we compared hemodynamic data of the T1 and T2 time-points with preinduction values, we found a decrease in the mean blood pressure and heart rate. However, this situation was similar between the groups and did not cause any problems in patients. From this information, we believe that there is no difference between the 2 laryngoscopes in terms of hemodynamic parameters.

Complications, such as tooth and soft tissue injury, bleeding, palatal perforation, or postoperative sore throat, may occur during intubation. There may be blind spots during the use of VLs. Complications may occur as a result of directing the endotracheal tube towards these blind spots [22]. Hoshijima et al. [15] claim that the VL enables less lifting force to be applied to the laryngeal structures and may cause less intra-oral damage. However, reports on this issue are contradictory [23–25]. In our study, we could not detect any difference between the 2 types of laryngoscopes in terms of complications. This may be because the intubation procedure is performed by an experienced anaesthesiologist on both types of laryngoscopes, and all patients except 1 intubated on the first try.

One of the limiting factors of our study is that the anaesthesiologist who applied the intubation procedure was not blinded to the laryngoscope model used, but this was not possible in practice. In addition, it was a single-centre study, and no intergroup comparisons were made regarding postoperative complications.

In conclusion, in patients undergoing bariatric surgery, intubation is expected to be difficult. Despite prolonging intubation time, better oropharyngeal and glottic images are obtained without causing hemodynamic changes with the McGrath VL. However, these advantages do not provide sufficient evidence for the McGrath VL to be used as the first choice.
